# Acute Kidney Injury in Monoclonal Gammopathies

**DOI:** 10.3390/jcm10173871

**Published:** 2021-08-28

**Authors:** Paolo Menè, Alessandra Moioli, Antonella Stoppacciaro, Silvia Lai, Francescaromana Festuccia

**Affiliations:** 1 Department of Clinical and Molecular Medicine, “Sapienza” University of Rome, 00189 Rome, Italy; 2 Division of Nephrology, Sant’Andrea University Hospital, Via di Grottarossa 1035-1039, 00189 Rome, Italy; moioli.am@gmail.com (A.M.); frarom73@yahoo.com (F.F.); 3 Division of Pathology, Department of Clinical and Molecular Medicine, “Sapienza” University of Rome, 00189 Rome, Italy; antonella.stoppacciaro@uniroma1.it; 4 Division of Nephrology, Department of Translational and Precision Medicine, “Sapienza” University of Rome, 00161 Rome, Italy; silvia.lai@uniroma1.it

**Keywords:** acute kidney injury, monoclonal gammopathies, multiple myeloma, immunoglobulins, light chains, hemodialysis

## Abstract

Monoclonal gammopathies (MG) encompass a variety of disorders related to clonal expansion and/or malignant transformation of B lymphocytes. Deposition of free immunoglobulin (Ig) components (light or heavy chains, LC/HC) within the kidney during MG may result over time in multiple types and degrees of injury, including acute kidney injury (AKI). AKI is generally a consequence of tubular obstruction by luminal aggregates of LC, a pattern known as “cast nephropathy”. Monoclonal Ig LC can also be found as intracellular crystals in glomerular podocytes or proximal tubular cells. Proliferative glomerulonephritis with monoclonal Ig deposits is another, less frequent form of kidney injury with a sizable impact on renal function. Hypercalcemia (in turn related to bone reabsorption triggered by proliferating plasmacytoid B cells) may lead to AKI via functional mechanisms. Pharmacologic treatment of MG may also result in additional renal injury due to local toxicity or the tumor lysis syndrome. The present review focuses on AKI complicating MG, evaluating predictors, risk factors, mechanisms of damage, prognosis, and options for treatment.

## 1. Introduction

An electrophoretically distinct, monoclonal β or γ globulin peak in serum [1,2] typifies monoclonal gammopathies MG). These could be subdivided into forms with undetermined significance (MGUS) or myeloma, either widespread at multiple bone-related sites (multiple myeloma, MM), or as a single “solitary” plasmacytoma (solid mass). Often individuals with MGUS abnormality of serum electrophoresis have no evidence of a systemic hematological disease nor organ damage such as heart failure, liver dysfunction, bone/skeletal alterations, or renal dysfunction. The prevalence of MGUS may vary from 3 to 7% in the general population, rising after the 5th decade of life. It is not uncommon that decades elapse without evidence of a clinically relevant hematologic disorder [2,3,4].

On the other hand, the onset of proteinuria, a sudden decrease in renal function or worsening over a few days of a pre-existing renal failure may reveal ongoing kidney damage in the context of the so-called monoclonal gammopathies of renal significance (MGRS). This subfamily of MGUS or MM was first defined in 2012 in a report by the International Kidney and Monoclonal Gammopathy Research Group [5,6]. Certain milder forms of MGUS without features of overt MM, previously referred to as “smoldering myeloma”, could well belong to this new category, since proteinuria and/or other signs of renal involvement are detectable. In MGRS, damage to the kidney could be massive, even though the bone marrow biopsy does not show features of “malignant” disease [6,7,8,9,10,11]. As an example, a non-myelomatous small clone releasing λ Ig light chains (LC) may result in renal amyloidosis, an accumulation of LC-derived fibrils along the kidney microvasculature. Glomerular deposition of amyloid substance results in a nephrotic syndrome (NS) with progressive renal failure, eventually leading to end-stage kidney disease [7,8,10,11,12]. LC or amyloid deposition occurs in other organs as well, including the heart and liver. Severe damage at this level may translate into organ failure, including fatal arrhythmias and/or liver failure [12].

Acute kidney injury (AKI) is not uncommon in patients with MG [13], including MGRS that do not fulfill the criteria for MM [5,6,10]. Causes include massive accumulation of MC paraproteins within glomeruli or renal tubules, which may become obstructed by crystals or luminal “casts” [9,10,11]. Hypercalcemia in patients with bone erosion in the context of MM may trigger pre-renal AKI through polyuria and dehydration, or perhaps by direct vasoconstriction of kidney vessels [13,14,15]. Glomerulonephritis (GNF) with immune complex or complement deposits containing LC paraprotein (proliferative glomerulonephritis with MC Ig deposits, PGNMID) has also been recently reported [10,16,17]. Several pharmacologic agents employed in the treatment of MG have adverse effects on the kidneys, including worsening renal function, occasionally presenting as AKI [18].

The present review, based on our 15-year clinical experience in this area, deals with the pathogenesis and treatment of AKI in MGRS, and the potential of tools such as the renal biopsy and high-cutoff hemodialysis membranes to support renal function while hematologic treatment becomes effective.

## 2. Acute Kidney Injury in MG: Pathogenesis and Clinical Presentation

AKI is diagnosed in more than 10% of all hospital admissions in most Western Countries [19,20]. However, the actual incidence is largely underestimated, due to inaccurate reporting by non-renal units at discharge and the frequent asymptomatic course in fragile/elderly patients who are not tested for renal function when outside of hospital. Our own experience of 24 histologically proven consecutive cases of MG points to a 1:6 ratio between AKI requiring HD treatment (Table 1) and the total number of patients with renal symptoms (Table 2). In a larger, multicenter collaborative cohort, the incidence of AKI episodes may be different, as a function of the length of observation and the choice of treatment. Episodes of asymptomatic, transient, and reversible renal dysfunction may be more frequent than expected. Among all causes of AKI, MG-related nephropathies are certainly a minority, as opposed to the leading causes, renal hypoperfusion/ischemia and renal toxicity of drugs and chemicals. Nevertheless, MG should always be considered in differential diagnosis, particularly among elderly individuals with hematologic abnormalities, unexplained hypercalcemia, proteinuria, and/or the NS [19,20,21]. The usual distinction in pre-renal, parenchymal, and obstructive causes applies to MG as well, although obstruction should be intended as intra-parenchymal rather than related to the extrarenal urinary tract. In other words, under most circumstances, precipitation of paraproteins within the tubular lumen accounts for obstruction of tubular outflow through what is referred to as “cast nephropathy” [22,23].

**Figure 1:**
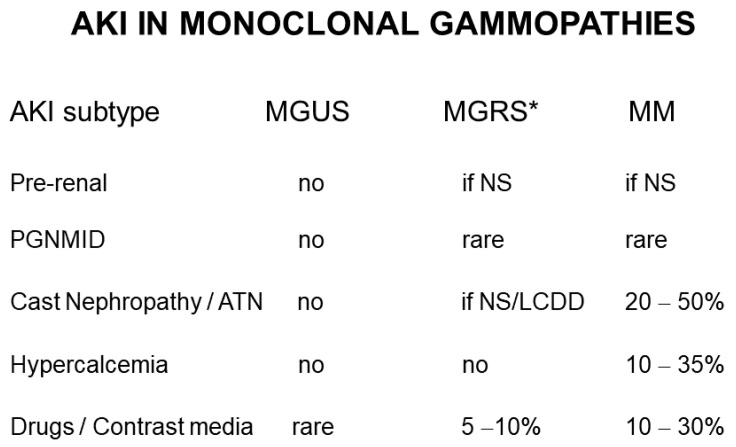
Major types of acute kidney injury complicating monoclonal gammopathies (MG), including forms of unknown significance (MGUS), forms with renal significance (MGRS), and hematologic malignancies such as multiple myeloma (MM). Prevalence data vary among different case series in the literature (range is given). Abbreviations: NS, nephrotic syndrome; PGNMID, proliferative glomerulonephritis with monoclonal Ig deposits; ATN, acute tubular necrosis; LCDD, light chain deposition disease. Drugs/agents potentially causing AKI include nonsteroidal anti-inflammatory drugs, radiological contrast media in the presence of significant blood levels of MC protein, or chemotherapy agents for MM as listed in Table 3. * In MGRS, NS may occur as a consequence of amyloidosis (without a bone marrow evidence of MM); similarly, LCDD with high levels of circulating MC protein may cause “cast” nephropathy.

Pre-renal AKI is common in NS, due to persistent hypovolemia related to the low levels of plasma proteins with resulting loss of oncotic forces recovering filtered fluids from tissue to the venous or lymphatic circulation (Figure 1). As a result, patients with massive glomerular proteinuria due glomerular LC deposition disease may become hypotensive. This is even more common in renal amyloidosis, which is typically complicated by widespread vascular damage and loss of vasomotor responses [12,24,25,26]. Persistent hypotension impairs renal blood flow, thus resulting in pre-renal failure. Prolonged hypoperfusion may evolve into thrombosis of glomerular capillaries and/or low tubular flow, which in turn could facilitate LC paraprotein cast formation within renal tubules [22,23]. Such scenario is that of a parenchymal type of AKI, involving glomerular ischemia and/or tubular necrosis.

PGNMID may actually present with features of parenchymal AKI, whenever proliferation, glomerular ischemia or necrosis occur in an extracapillary pattern, resembling a “crescentic” vasculitis [7,8,9,10,11,16,17]. Another pattern of functional renal impairment, which could eventually lead to AKI through renal ischemia, is one of renal edema, once again related to low oncotic force of plasma during the NS, induced by LC deposition disease (LCDD) or PGNMID (Figure 1). A large, swollen kidney at ultrasound or CT scanning may suggest renal intraparenchymal fluid accumulation, which could translate into elevated hydrostatic pressure within the organ due to a scarcely stretchable capsule [27,28]. The setting is similar to that induced by muscle ischemia following trauma, infection, or massive exercise with tissue swelling, the so-called “compartmental syndrome”. Poorly distensible peri-muscular fasciae are responsible of serious ischemia related to injuries to the forearms or legs, as an example [29]. The renal condition has been referred to as “nephrosarca” in some studies, echoing the “anasarca” setting of serosal edema in NS, cardiac failure, or decompensated cirrhosis of the liver [27,28,29]. AKI may, thus, well occur in NS due to kidney edema and resulting local ischemia (Figure 1).

As mentioned earlier, hypercalcemia is not infrequent in MM with extensive bone involvement [14,15]. Enhanced osteoclast activity triggered by cytokines or chemokines released by proliferating plasma cells results in bone reabsorption independent of hyperparathyroidism. Elevated circulating levels of Ca^2+^ have vasoconstrictor effects on kidney vasculature, thus reducing renal blood flow. Moreover, hypercalcemia has direct inhibitory effects on water transport across distal tubules, inducing in animal models autophagic degradation of aquaporin-2 (i.e., ADH-inducible water channels) in collecting duct epithelia. As a result, polyuria ensues, which is not due to the osmotic effects of Ca^2+^ themselves, as initially believed, but rather to such impaired water reabsorption (see below, section on “Effects of hypercalcemia on kidney function”) [30,31]. Dehydration could be massive for serum Ca^2+^ levels above 12–14 mg/dL, with pre-renal AKI (Figure 1). Chronic hypercalcemia may also induce medullary deposition of Ca^2+^ salts, or nephrocalcinosis, with longer-lasting effects on renal function.

One last potential cause of AKI in MG is renal vein thrombosis, once again a known complication of the NS [32,33]. The hypercoagulability often encountered in NS is due to the loss of coagulation inhibitors through proteinuria, particularly fibrinolysin. It translates in some individuals into deep vein thrombosis, giving rise to organ injury and occasionally pulmonary embolism, more frequently in patients with membranous glomerulopathy. Bilateral occlusion of renal veins (or vena cava above the confluency of renal veins) may result in AKI, usually associated with bilateral flank pain and gross hematuria [32,33].

## 3. Acute Kidney Injury: Risk Factors and Their Relationship to MG

MG may occur in the context of other comorbidities which impair renal function or weaken compensatory responses of the kidney to vascular injury. Advanced age, functional overload to the kidney due to the metabolic syndrome, diabetes mellitus, heart failure, and hypertension are just a few of a long list of conditions that could amplify the effects of paraprotein deposition in the vasculature or tubulo-interstitial compartments of the kidney. Pre-existing chronic renal failure or the unstable glomerular filtration rate often seen in chronic heart failure are predictors of AKI in individuals with MG. Antihypertensive or “renoprotective” treatment with ACE-inhibitors or angiotensin II (ANG II) receptor antagonists may amplify the effects of MC protein deposition in kidneys whose glomerular filtration is reduced to same extent, particularly in elderly subjects with atherosclerosis/vascular damage. The same concept applies to treatment with nonsteroidal anti-inflammatory drugs (NSAIDs) inhibiting cyclooxygenase, reducing blood flow while enhancing tubular Na^+^ and water reabsorption. These agents are often used for the treatment of osteoarthritis, particularly among elderly patients, who are more prone to develop MC components in their serum [34]. Bone infiltration and spontaneous fractures among MM patients are another leading cause of treatment with NSAIDs. Unfortunately, inhibition of constitutive cyclooxygenase in kidney vessels and tubules exposed to toxic paraproteins often results in further impairment of renal hemodynamics and tubular transport, thus amplifying the risk of AKI [34]. Thus, a careful pharmacologic anamnesis should always be obtained whenever a MG is identified, offering advice on avoiding the renal adverse effects of certain classes of drugs.

## 4. Handling of MC Proteins by the Kidney—Tubular Toxicity of Filtered Proteins

MC proteins are quite heterogeneous, based on their molecular structure (κ or λ, light or heavy chains, anionic vs. cationic net charge, etc.) [35,36]. Yet, the common response to filtering into the urinary space is an attempt to reabsorb the MC proteins. The process takes place mostly at the level of the brush-border of proximal tubules and is common to most filtered amino acids, peptides, and proteins, independent of their molecular structure [37]. Plasma membrane receptor/carrier molecules such as megalin, cubilin, and the related transmembrane protein, amnionless, bind soluble, or insoluble free proteins, internalizing them through endocytosis into the cytosol of tubular epithelial cells [38,39]. An enzymatic digestion then takes place within cytosolic vacuoles or “phagosomes”, lysosomal units engulfing foreign material, reducing proteins into smaller peptides, or individual amino acids. Acidification of the vacuoles is critical to processing reabsorbed proteins and relies upon a vacuolar ATPase or H^+^/“proton pump”, in turn enabled by a Cl^−^ channel balancing the electrogenic consequences of H^+^ transfer [38,39]. Since this mechanism is aimed at reabsorbing small loads of proteins escaping the glomerular permselectivity barrier (e.g., insulin, peptide hormones, trace albumin), massive proteinuria as in the case of MC LC is likely to overload tubular epithelial cells, resulting in toxicity and direct cell injury [40]. This is relevant to the so called “cast nephropathy” discussed in the next paragraph, a frequent complication of MGRS and/or MM [22,23], as well as of many proteinuric disorders leading to the nephrotic syndrome.

## 5. Tubular Obstructive Nephropathy

“Casts” are proteinaceous or crystalline aggregates that occlude the tubular lumen when the filtered mass of proteins or crystals exceeds reabsorption by epithelial cells [17,22,23,41,42,43,44]. Intense water reabsorption such as during high-dose diuretic therapy or dehydration for intercurrent disorders (gastroenteritis, colitis, water deprivation for any reason) enhances protein accumulation by reducing urine flow and increasing luminal protein concentration. This condition is not unique to MGRS or MM but occurs in massive nephrotic syndrome or crystal precipitation as well (e.g., urates or phosphates in the tumor lysis syndrome, polyethylene glycol intoxication, etc.) [45,46,47,48,49,50,51].

Data from MG series quite consistently describe histologic evidence of cast nephropathy in about 65% of cases with a relevant urinary paraprotein output. An autopsy series demonstrated tubular obstruction by monoclonal LC in nearly all cases, even when renal failure is not reported during the final course of disease [47,48,51]. Most likely, surviving functional nephrons could compensate, at least to a certain extent, the degree of obstruction.

“Cast nephropathy” should always be confirmed by a renal biopsy whenever a patient with MGRS experiences a sharp reduction in urine output or frank oliguria, whereas creatinine and BUN levels rise in a short time of 1 to 2 weeks (Figure 1) [47,48]. It should be noted that a decrease in 24-hour urine volume may not be appreciated until enough renal mass is functionally impaired, due to reduced water reabsorption by the surviving, non-obstructed renal tubules. If for any reason a kidney biopsy could not be obtained, empirical hydration and urine alkalinization by NaHCO_3_ infusion or K/Mg-citrate should be undertaken, in order to hasten clearance of the filtered load of paraprotein.

## 6. Crystal Precipitation of MC Proteins

A rare clinical finding in renal biopsies of patients with MGRS is a crystal-storing nephropathy with deposition of rod-shaped or rhomboid crystals of LC within podocytes and/or proximal tubular epithelial cells [17,43,44]. More than an endoluminal accumulation of crystals with obstructive nephropathy, as seen in the tumor lysis syndrome with urates or sodium phosphate crystals, these MC protein crystals (most often IgGk) tend to cluster within the cytoplasm of glomerular or tubular cells, likely engulfed in phagolysosomes. Nevertheless, tubular casts containing MC protein crystals can also be found on microscopic examination of the urine. The basic histology of nearly 15 reported cases in the literature resembles a focal glomerulosclerosis, consistent with the clinical presentation of proteinuria occasionally evolving into a nephrotic syndrome with rapidly progressive renal failure [43].

## 7. The Effects of Hypercalcemia on Kidney Function

First described in 1921, hypercalcemia of malignancy occurs in up to 20% of all patients with advanced cancer and generally conveys a poor prognosis [52,53]. MM is one of the more common cancer diagnoses associated with hypercalcemia, resulting from local osteolytic bone resorption [54]. There are three principal mechanisms of hypercalcemia in cancer patients. Secretion of parathyroid hormone (PTH)-related protein (PTHrP) by tumor cells—known as humoral hypercalcemia of malignancy—accounts for 80% of cases and occurs most commonly with squamous cell tumors [55]. Upon binding to PTH receptors in bone and kidney, PTHrP regulates bone resorption and renal handling of Ca^2+^ and phosphate [53]. Another 20% of cases arise directly from osteolytic activity at sites of skeletal metastases. Breast cancer, MM, and lymphomas commonly cause hypercalcemia via this mechanism. Rarely, hypercalcemia may result from direct tumor secretion of vitamin D, which has been described in association with certain lymphomas or from ectopic tumor secretion of PTH [55].

Hypercalcemia is defined as a serum Ca^2+^ level above the upper limit of the normal reference range (usually 10.5 mg/dL) and can be categorized as follows: mild, from 10.5 to 11.9 mg/dL; moderate, from 12 to 13.9 mg/dL; severe, >14 mg/dL [56,57,58]. The clinical features of hypercalcemia include nausea, vomiting, lethargy, renal failure, and coma. Symptom severity depends not only on the degree of hypercalcemia, but also on the rapidity of onset and the patient’s baseline neurologic and renal function [55]. Hypercalcemia is most commonly caused by increased bone resorption with release of Ca^2+^ from bone and the inadequate ability of the kidneys to manage higher Ca^2+^ levels [59,60]. Renal manifestations of hypercalcemia consist of nephrogenic diabetes insipidus with resulting polyuria, renal vasoconstriction, distal renal tubular acidosis, and, in more chronic situations, nephrolithiasis, tubular dysfunction, and chronic renal failure [60]. In MM, hypercalcemia may potentiate other AKI etiologies: Ca^2+^ promotes direct afferent arteriolar vasoconstriction and also leads to volume depletion from excessive renal Na^+^ and water loss. The mechanism is two-fold: Ca^2+^ causes Na^+^ wasting at the loop of Henle by activating the Ca-sensing receptor. It also leads to renal water losses by blocking AVP activity in the distal nephrons via targeted autophagic degradation of aquaporin-2 and some 15 other proteins associated with cytoskeletal protein binding and cell–cell junctions at the level of inner medullary collecting duct [30,31]. Rarely, severe hypercalcemia may cause AKI via intratubular calcium-phosphate deposition [13].

The primary therapy of hypercalcemia targets the underlying malignancy. The type and timing of therapy are determined by the severity of hypercalcemia and associated symptoms. In mild, asymptomatic hypercalcemia, treatment could be delayed until the laboratory tests have been completed and diagnosis has been made. In moderate to severe hypercalcemia, when severe renal and neurologic symptoms are present, along with EKG changes, treatment should be started immediately [14].

Patients with hypercalcemia are often dehydrated from the outset due to poor intake secondary to nausea, vomiting, altered mental status, and hypercalcemia-induced nephrogenic *diabetes insipidus* [30,31]. Furthermore, volume contraction in itself compromises renal handling of Ca^2+^ due to hypovolemia-mediated increased reabsorption in the kidneys [60]. Volume expansion with isotonic saline is the initial treatment of choice to restore renal perfusion and to increase renal Ca^2+^ excretion. Usually, a bolus of 1–2 L of isotonic saline is administered followed by maintenance fluids at a rate of 100–150 mL/hour titrated to ensure a urine output of 100 mL/h. The addition of furosemide to promote calciuresis is generally not recommended and should be reserved for patients with congestive heart failure and symptoms of volume overload or in the case of oliguric renal failure [61].

The second option that should be considered in the management of severe hypercalcemia is calcitonin intramuscular or subcutaneous administration. The typical dose range is from 4 to 8 UI/Kg every 6–12 h. The duration of administration is usually limited to 48 h. Calcitonin seems to act via the inhibition of osteoclast activity and an increase in renal Ca^2+^ excretion.

Bisphosphonates comprise a group of medications that are analogs of natural pyrophosphate, which is an essential part of bone. The mechanisms of action include the impairment of osteoclast-mediated bone resorption, the arrest of osteoclast development, osteoclast apoptosis, and a decrease in osteoblast apoptosis [60].

Zoledronic acid and pamidronate are the bisphosphonates more commonly used for the treatment of hypercalcemia of malignancy. Zoledronate is the most potent compound in this class, and the typical dose is 4 mg administrated iv over 15–30 min. Unfortunately, it has been associated with nephrotoxicity; a dose reduction according to the glomerular filtration rate (GFR) is recommended as follows: GFR 50–60 mL/min, 3.5 mg; 40–49 mL/min, 3.3 mg; 30–39 mL/min, 3 mg. Zoledronate should not be administered when GFR drops <30 mL/min [14]. Pamidronate doses range from 60 mg to 90 mg administered iv over 2–6 h; for a GFR between 30 to 60 mL/min the appropriate dose is 30 mg iv; pamidronate is not recommended for a GFR <30 mL/min [14,15].

Corticosteroids are also employed due to their important clinical effects in the setting of hypercalcemia associated with MM and other hematological malignancies. Glucocorticoids inhibit 1α-hydroxylase conversion of 25-hydroxyvitamin D into 1, 25-dihydroxyvitamin D, therefore reducing intestinal Ca^2+^ absorption. They also inhibit bone resorption by osteoclasts by decreasing the tumor production of locally active cytokines, in addition to having direct tumorlytic effects. Steroid schedule usually employs hydrocortisone 200–400 mg/day for 3–4 days, followed by prednisone 10–20 mg for 7 days or prednisone 40–60 mg/day for 10 days; the expected decrease in serum Ca^2+^ levels may reach >3 mg/dL within 7 days after initiating therapy [14].

Denosumab is a human monoclonal antibody to RANKL, inhibiting osteoclast activity and bone resorption. Denosumab has been shown to be effective in hypercalcemia refractory to bisphosphonates. A further advantage of denosumab over bisphosphonates is that it is not removed by the kidneys and has been shown to improve renal function in patients with MM and hypercalcemia [62]. The typical dose of denosumab is 120 mg subcutaneously and should be repeated no earlier than 1 week following the first administration. The hypocalcemic effect is typically seen within 2–4 days of administration [63]. Ca^2+^ reduction may be more pronounced in patients with renal failure, so that dose reduction is recommended to avoid hypocalcemia [62].

For patients with acute hypercalcemia and significant AKI (especially in the setting of oliguria and cardiac disease), saline-induced diuresis may not be feasible and may lead to volume overload. In these circumstances, hemodialysis (HD) using a low-Ca^2+^ dialysate (1.25 mmol/L) is a safer option [64].

## 8. AKI Related to Chemotherapy of MGRS/Multiple Myeloma

The impact of treatment for MGRS or MM on renal function is not easily assessed, since patients with such hematologic diseases are often already showing signs of renal damage due to the burden of paraproteins or related disorders, such as hypercalcemia [14,15,18]. Usually, renal failure is progressive, thus it is not easy to dissect out renal injury related to disease itself rather than the adverse effects of treatment. Therefore, it is of utmost importance to carefully evaluate renal function at the time of initiating treatment, both in order to choose a treatment regimen that could be well tolerated by patients with failing kidneys, and to avoid attributing AKI to toxicity of drugs, when it rather results from the direct harmful effects of MC paraproteins over months of renal accumulation [18].

Table 3 summarizes the wealth of novel agents that have been introduced in the treatment of MM and also certain forms of MGRS in the past 10–15 years. They are gradually replacing conventional chemotherapy agents such as cisplatin, melphalan/alkeran, cyclophosphamide, and anthracyclines, which are often poorly tolerated and associated with serious renal complications [65,66,67,68,69,70,71,72]. These include electrolyte disorders (all), tubular toxicity and necrosis (cisplatin), AKI (melphalan), hemorrhagic cystitis (cyclophosphamide [73]), and glomerular proteinuric disease (anthracyclines [74]). Newer agents belonging to the classes of immunomodulators and proteasome inhibitors generally have a better safety profile. Nevertheless, there have been numerous reports of AKI (lenalidomide, pomalidomide), occasionally linked to thrombotic microangiopathies (bortezomib, carfilzomib). On the other hand, HDAC or SLAMF7 inhibitors and anti-CD38 monoclonals seem to have lesser effects on the kidney, although their diffusion is still far lower than immunomodulators or proteasome inhibitors [18]. Early phase 1–3 trials with BRAF inhibitors, immune checkpoint inhibitors, MEK inhibitors, and anti-KIR inhibitors have again shown some tendency to trigger AKI or interstitial nephritides. Overall, lenalidomide, everolimus, and bortezomib are by far responsible for the majority of cases of AKI among all agents currently employed. This does not indicate a worse safety profile; however, as the three agents are also the mainstay of current therapy of MM worldwide, particularly the proteasome inhibitor, bortezomib. Moreover, many reports of renal “toxicity” by MM therapeutics are not backed by renal biopsies, so that it is not easy to distinguish drug-related adverse events from disease progression (Figure 1). Thus, careful evaluation of renal function and electrolyte balance is mandatory for all patients being treated for MM or related gammopathies, under the direct supervision of an onco-nephrologist with experience in the quickly growing field of anti-cancer treatments [75].

## 9. The Burden of Age: Epidemiology of AKI Related to MG

Incidence and prevalence of MG tend to increase with age, so that the vast majority of cases are seen among subjects in their 6th decade of life or older [1,2,3,4,7,8]. In our own experience of MG (Table 1 and Table 2), 24 patients had a mean age of 62.5 ± 9.1 years (range 42–76) at the time of renal biopsy. Of these, four cases out of 24 histologically proven MG presented as AKI (Table 1). Their mean age was definitely higher, at 73.2 ± 1.8 yrs. This suggests that the workup of patients with rapidly progressive (RP) renal failure or unexplained AKI in this age group should always include assessment of serum Ca^2+^ levels (none of our cases had hypercalcemia) and, obviously, the possible occurrence of a MGRS/MM. Serum electrophoresis, a search for a Bence-Jones proteinuria and possibly measurement of serum FLC levels are the definitive tools to implicate a MGRS in the pathogenesis of AKI. On the other hand, a reduced renal reserve or co-morbidities in the elderly make this age group prone to develop kidney failure once stressors such as paraprotein deposition and/or aggressive treatment of MM are applied. Age is also a key predictor of the outcome of renal disease once the MG diagnosis is established and treatment is started, since the likelihood of a full or partial recovery decreases with time, due to the limited residual nephron mass.

## 10. Renal Biopsy: Linking Persistent Urinary Abnormalities and AKI or Progressive Renal Failure to MGRS

Renal biopsy is the ultimate tool to obtain an accurate diagnosis of MGRS [11,76,77,78]. Its relevance exceeds the actual identification of the renal lesion leading to urinary abnormalities or AKI. Its limitations are related to frequent contraindications such as advanced age, renal cysts, the need for anticoagulant therapy following cardiac surgery, and the presence of other comorbidities that impact on the kidneys, such as diabetes mellitus. Diabetic microvascular disease or frank diabetic nephropathy could obscure the role of MGRS as a cause of progressive renal failure. Actually, an MGUS usually followed through a “watchful wait” approach may be recognized through the renal biopsy as a systemic disorder with impact on target organs. It will, thus, deserve treatment, even if it does not fulfill the criteria for a diagnosis of MM or other major hematological disease.

On the other hand, reliable serologic or urinary markers of ongoing renal damage by a MGRS, thus avoiding the need for a renal biopsy, are yet to be found. As an example, exosomes, macrovesicles containing precursors of the paraproteins delivered to the kidney and potentially harmful, could be recovered from the urine by ultracentrifugation [79,80,81]. The technique is very accurate and provides an excellent insight into the mechanisms by which amyloid substance is deposited in the kidney, as an example. However, it is cumbersome and labor-intensive, requesting proteomics tools, including GM spectrometry liquid chromatography [82,83]. The renal biopsy is instead easy to obtain, less invasive than a myocardial or liver biopsy, and provides useful information within few days, if not already at bedside when snap-frozen sections are examined by hematoxylin/eosin prior to forwarding tissue sample to the Path laboratory. This should always be done when AKI of unclear etiology requires rapid choices between renal replacement therapy (i.e., HD/renal apheresis in a cast nephropathy) or aggressive pharmacologic treatment, as may be appropriate in a rapidly progressive GNF/PGNMID [7,8,9,10,11,16,17].

In order for a renal biopsy to be fully diagnostic, light microscopy with hematoxylin/eosin, PAS, and silver methenamine or Masson’s trichrome should be performed [11,76,77,78], followed by immunofluorescence screening for κ and λ LC. Polarized light microscopy of Congo Red or Thioflavin T-stained sections is also quite useful to detect AL amyloidosis. Since these stains are technically delicate and operator-dependent, the results should be better backed by transmission electron microscopy (EM) [83]. The typical 7.5–10 Å random-oriented fibrils within intramural and perivascular deposits are a highly sensitive and specific marker for AL amyloidosis [12,25,66,78,80,81,82,83]. Other forms of MC protein deposition within glomerular capillaries can be resolved on the basis of EM, such as fibrillary GN, cryoglobulinemic GN, C3 nephropathy, or immunotactoid glomerulopathy [84,85,86,87,88,89,90,91]. These nephropathies share a mesangial or mesangio-proliferative pattern on light microscopy, with typically Congo Red-negative deposits that do not resemble amorphous immune complexes, as seen in GN. The EM appearance is that of randomly oriented fibrils with a thickness of 13–29 nm or, rather, orderly bundles of microtubules with an average diameter ranging from 20–30 nm (cryoglobulins) to 10–90 nm (immunotactoids) [84]. Fibrillar or immunotactoid deposits can be observed also in lymphoproliferative disorders, such as B-cell lymphomas [78,84], while cryoglobulins span across membranoproliferative GN, mixed essential cryoglobulinemia, or systemic lupus erythemathosus (SLE) [86,87].

As mentioned earlier, rare forms of “crystalline” glomerulopathies have been described, featuring inclusions within the cytoplasm of podocytes and parietal glomerular epithelial cells, along with proximal tubular deposition [43,44]. Crystals of LC are thought to occur because of a unique resistance of certain paraproteins to proteolysis within lysosomes [42,43,44]. “Crystalglobulins” have also been reported within the glomerular capillary walls; these deposits may be also found in the skin microcirculation, resulting in ulcers and purpuric lesions [7,9,92].

Deposits of LC or HC within the capillary walls and the mesangium resemble nodular diabetic glomerulosclerosis (Kimmelstiel–Wilson glomerulopathy), indicating an LCDD [76,77,78]. This pattern is usually associated with heavy proteinuria progressing to the NS, along with early loss of function that may even occur within a few months or occasionally as a rapidly progressive renal failure. Glomerular LC deposits are usually faintly PAS-positive, unlike the brisk staining of diabetic glomerulopathy. Diagnosis is based on immunofluorescence, which shows the selective κ or λ LC nature of the deposits, and by EM, showing the coarse aspect of deposits as opposed to the extracellular collagenous matrix of diabetic nephropathy [8,48]. Tubulo-interstitial LCDD is probably more common; although, renal biopsies are not performed in the majority of MGRS without renal failure or high-grade proteinuria [46,47,48]. Early clinical signs of tubular involvement can be grouped into the acquired “Fanconi syndrome”, i.e., altered acidification mechanisms with tubular acidosis, tubular proteinuria (non-selective, mild, usually <1.5 g/day), glycosuria, aminoaciduria, low-serum phosphate, and uric acid due to impaired tubular reabsorption [49]. Selective defects can also be seen, based on the extent of tubular damage.

Tubular abnormalities are quite apparent in renal biopsy, beyond the luminal positivity of IF for κ or λ LC. Tubules appear enlarged, with thickened and tortuous basement membranes, lined by deposits on the outer side. Cells undergo various processes of apoptosis, “blebbing”, and vacuolization, with a decrease in viable cell numbers; loss of the brush-border can be noted in the proximal tubule. In advanced stages, detachment of the epithelial monolayer occurs, along with clumps of cellular debris amidst amorphous proteinaceous material, i.e., precipitated LC [46,47,48,49], within the tubular lumen. The resulting “cast nephropathy” shares features of acute tubular necrosis (ATN) [50,51].

## 11. Treatment of AKI by Renal Apheresis: High-Cutoff Membrane Hemodialysis

Up to 40% of all patients with MM display kidney damage of mild or moderate grade, while severe acute kidney injury (AKI) occurs in approximately 9% of cases [93,94,95,96,97]. Recovery of renal function is a predictor of improved survival, although AKI requiring hemodialysis (HD) is often irreversible [97,98]). Besides the need for HD as a renal replacement therapy, in view of tubular obstruction and the known direct tubular toxicity of FLC, the goal of any therapy for MG should also aim at reducing exposure of the kidney to FLC [98,99,100,101,102]. A linear relationship has been described between the probability of renal recovery and both the degree and speed of FLC reduction [101]. The half-life of circulating FLCs is 3–6 h in subjects with normal renal function, but it shows an inverse correlation with GFR, as the kidney is the main FLC catabolism site. Thus, FLC half-life increases up to 10-fold in patients with CKD stage 5 [101]. A reduction of more than 50% of serum FLC concentration is needed to achieve renal rescue [102]. Rapid inhibition of FLC production from plasma cell clones by chemotherapy might not yield an immediate reduction in serum concentration, since the volume distribution of FLC is quite large, well beyond the intravascular compartment, so that a refilling of the general circulation by FLC accumulated in soft tissues and extravascular spaces readily occurs [103,104]. Therefore, the kidney might be exposed to elevated levels of FLC for several weeks, despite initiation of chemotherapy [105]. The removal of FLC may play a complementary role to chemotherapy in obtaining a faster kidney response [104,106,107]. In this scenario, the use of extracorporeal techniques for the treatment of MK provides direct and rapid support to this purpose, helping to rapidly clear FLC from both intravascular and interstitial compartments [104].

Therapeutic apheresis (TA) is an extracorporeal blood purification method employed in treating renal diseases caused by the accumulation of immune complexes, allo- or autoantibodies, and cryo- or immunoglobulins in the patient’s plasma. Removal of those substances by way of apheresis promises either a total remission (apheresis as a primary treatment) or improved results of an immunosuppressive treatment (apheresis as a supporting treatment, [108]). The first attempts at a therapeutic application of plasmapheresis were conducted in 1952 on patients with a hyperviscosity syndrome in the course of MM [109]; plasmapheresis was the only extracorporeal technique capable of removing circulating FLCs until 2005. Several studies have been conducted to evaluate its efficacy. Nevertheless, there are only three published randomized and controlled clinical trials, and all of them failed to demonstrate a therapeutic effect of plasmapheresis on MM renal dysfunction [110] However, removal of FLC by plasmapheresis has usually been considered inefficient due to the relatively limited volume of plasma as compared to the FLCs high volume of distribution [111]. In 2008, Leung et al. published a retrospective study on plasmapheresis considering renal biopsy findings and FLC reduction. The study clearly illustrates the importance of the histological diagnosis and the relationship between FLC reduction and clinical outcomes in myeloma cast nephropathy [102].

In 2005, a new generation of HD membranes known as “protein leaking membranes” were designed as an alternative way to provide greater clearances of high MW substances involved in uremia, which are not removed by high-flux membranes [112]. The so called “high cut-off membranes” owed their name to their wide pore size, which enhanced the MW cut-off to 50–60 KDa [113]. HD with high-cut off dialyzers (HCO-HD) has been shown to achieve a significant reduction in post-dialysis serum FLC levels. Indeed, HD with a HCO-1100 dialyzer, which has a membrane surface area of 1.1 m^2^, was effective in reducing both κ and λ FLC levels. However, by using 2 HCO-1100 dialyzers in series and, therefore, doubling the membrane surface area, there was a greater increase in FLC clearance and FLC reduction ratios [114,115]. Subsequently, it was shown that patients who received effective chemotherapy regimens and adjunctive HCO-HD had a much better renal recovery rate than historical controls [101,108,116]. The most relevant studies on FLC removal by using HCO filters were published in 2007 and 2008 by the same group [103,105]. One of the key conclusions reached in these studies is that HCO-HD could be useful in accelerating the decrease in serum FLCs yielded by effective chemotherapy. Therefore, without a concomitant decrease in the FLC production rate, extracorporeal depuration was found to generate only a transient decrease in FLCs followed by a “rebound” thereafter [106,115]. Two subsequent studies [101,104] examined whether early reduction in serum FLC was associated with better outcomes, and if there was a specific FLC reduction threshold that granted the renal improvement. The authors found that FLC reduction on day 21 was associated with 60% of renal recovery and 80% of HD independence. Finally, two randomized clinical trials were conducted to prove the efficacy of HCO-HD as an adjuvant therapy for HD-dependent AKI in MM patients receiving concomitant chemotherapy. One of them, the European Trial of Free Light chain removal by extended HD in cast nephropathy (EuLITE), failed to show improved clinical outcomes for patients with de novo MM and cast nephropathy who required HD for AKI who received a bortezomib-based chemotherapy regimen, relative to those receiving standard high-flux HD (HF-HD) [116]. These results did not support proceeding to a phase 3 study for HCO-HD in these patients [116,117]. Furthermore, high cost, elevated protein leakage requiring albumin replacement, and calcium/magnesium wasting are the major drawbacks limiting its utilization [118]. It should be noted that prior to these reports the International Myeloma Working Group (IMWG) in their 2011 recommendations on myeloma-related renal impairment had stated that the current data support the use of HCO-HD in AKI HD dependence secondary to MCN with an evidence grade B [96].

To enable the clearance of “middle molecules” with MW between 15 and 60 KDa without the loss of albumin, the distribution of the pores within the dialysis membranes was re-designed to a tighter distribution. The medium-cutoff membranes (MCO) dialyzers use this new distribution of pore. In clinical practice, these membranes should provide an effective clearance of large molecules without excessive albumin loss [119]. The REMOVAL-HD study demonstrated safety and efficacy of MCO in comparison with high-flux dialysis sessions [119].

## 12. Treatment of AKI by Renal Apheresis: Hemofiltration with Endogenous Reinfusion

Enhancing convective clearance through hemodiafiltration (HDF) has been shown to be more efficient than conventional HD to reduce the levels of middle-size molecules [120]. Hemofiltration reinfusion (HFR) is a form of hemodiafiltration (HDF) in which the replacement fluid is constituted by ultrafiltrate from the patient “regenerated” through a cartridge containing a hydrophobic styrene resin that utilizes separated convection, diffusion, and adsorption. A two-stage filter is applied that consists of a high-flux polyethersulfone unit in the first convective stage (membrane cut-off 42 KDa) and a low-flux polyethersulfone filter in the second diffusive stage to enhance complete separation of convection from diffusion. In the convective phase of the first stage, pure ultrafiltrate (plasmatic water) passes through a sorbent cartridge containing 40 mL of hydrophobic styrene resin constituted by numerous pores and channels that add to its extensive surface area (~700 m^2^/g). Treatment is performed on a Flexya monitor (Medtronic S.R.L., Mirandola, Italy) equipped with software that automatically determines an optimal ultrafiltration flow rate (Q_uf_). The sorbent cartridge has a high affinity for several uremic toxins and middle molecules, including β2-microglobulin, homocysteine, angiogenin, leptin, parathyroid hormone (PTH), several chemokines, cytokines, and immunoglobulin. Urea, creatinine, uric acid, Na^+^, K^+^, Ca^2+^, phosphate, and bicarbonate are not adsorbed and remain unchanged after passage through the cartridge. They can be managed by diffusion during the second stage of filter. Thus, the “regenerated” ultrafiltrate is an endogenous ultrapure replacement fluid with a physiologic content of bicarbonate [121,122].

A retrospective analysis has shown the depurative superiority of convective over diffusive strategies in MM patients [123]. This technique is used in HD patients for its high protein-bound toxin adsorption capacity without removal of albumin [111,124,125]. HFR-SUPRA could provide efficient clearance of FLC in view of its molecular size cut-off, which theoretically allows FLC passage, and of the high affinity of the adsorptive cartridge [126].

We have used HFR-SUPRA in five consecutive patients with MM and AKI along with i.v. dexamethasone and followed within one week by a Bortezomib-based chemotherapy (Table 4) [127]. In each session, we observed an average decrease in FLC between 37.7 ± 21.3% and 57.0 ± 17.7% for κ chains and between 48.8 ± 8.7% and 71.6 ± 5% for λ. Five sessions of HFR-SUPRA on alternate days allowed a fast and stable reduction in serum FLC levels of about 46.5% for κ and 60.2% for λ), despite the serum rebound due to refilling from tissue deposits (Table 4). Furthermore, albumin wasting has not been neither statistically or clinically relevant during the whole treatment (−3 to 7%). From a prognostic point of view, recovery of renal function is a key factor: two of our patients achieved complete remission, while three patients who remained HD-dependent were older and had a longer history of misdiagnosed or untreated MM symptoms [127].

## 13. Conclusions

In summary, MG are an area in which the collaboration of hematologists and nephrologists is highly recommended and quite effective. Early diagnosis is necessary, so that a thorough evaluation of renal function followed by a renal biopsy is always recommended as a standard approach to MGRS. Notably, most patients are seen initially due to renal abnormalities, and a MC component often appears as an unexpected finding during a first laboratory screening. The availability of renal replacement/aphaeretic techniques may initially help as a bridge to an effective response to pharmacologic treatment, which may require weeks to months [41,127]. Clone-directed therapies and avoidance of all medications that may negatively impact on renal function could greatly benefit patients with MGRS, whose prognosis may be more favorable than in overt MM, dramatically reducing the risk of eventual renal or cardiac failure. Besides the obvious goal of preserving renal function as far as quality of life of these patients is concerned, the issue is also critical to enable various novel therapeutic options for hematological malignancies, including new molecular tools, monoclonal Abs, and/or stem cell transplantation [68,69,70,71,72].

## Figures and Tables

**Table 1:** AKI in 4 consecutive patients with histologically proven renal injury related to monoclonal gammopathies.

Patient	Age	M/F	eGFR, mL/min	uProt, g/day	Diagnosis
M.M.B.	72	F	10.6 (pre-HD)	5.0	AKI, AL Amyloidosis, IgG λ
R.D.C.	72	F	9.5 (pre-HD)	7.5	AKI, AL Amyloidosis, IgG λ
B.M.	73	F	11.9 (pre-HD)	5.0	AKI, Cast nephropathy, IgG k
A.P.	76	M	12.4 (pre-HD)	10.0	AKI, Cast nephr., AL Amyl., IgG λ
	73.2 ± 1.9		11.1 ± 1.3	6.9 ± 2.4	

Data are expressed as mean ± SD. Abbreviations: eGFR, estimated glomerular filtration rate (CKD-EPI equation); uProt, proteinuria; MM, multiple myeloma; HD, hemodialysis.

**Table 2:** Clinical characteristics in 20 consecutive patients with histologically proven renal injury related to monoclonal gammopathies and stable renal function or chronic progression.

Patient	Age	M/F	eGFR, mL/min	uProt, g/day	Diagnosis
G.C.	65	M	33.1	5.2	LCDD, IgM λ
E.D.B.	72	M	30.4	1.1	LCDD, IgM λ
L.F.	66	F	25.1	8.8	LCDD, IgG k
I.D.T.	71	F	50.7	13.2	LCDD, IgM λ
G.G.	60	F	32.3	4.1	LCDD, IgG k
M.L.	45	M	96.7	8.8	LCDD, IgG k
G.M.M.	42	F	107.1	2.2	LCDD, IgG k
M.C.S.	52	F	65.3	5.8	LCDD, IgG k MM
D.S.	64	M	45.8	8.5	LCDD, IgG k MM
L.B.N.	51	F	36.6	1.9	AL Amyloidosis, IgG λ mMM
V.T.	62	M	26.3	1.2	AL Amyloidosis, IgA λ
T.A.	64	M	52.2	11	AL Amyloidosis, IgA λ mMM
G.C.	54	M	108.5	5	AL Amyloidosis + FibGNF, IgM l
R.C.	68	M	100	4.8	AL Amyloidosis, IgA λ
R.G.	54	F	160.2	3.1	AL Amyloidosis, IgM λ MM
M.L.	59	M	94	5.3	AL Amyloidosis, IgM λ
E.L.C.	70	M	84.3	4.6	AL Amyl., B-cell lymphoma, IgG k
P.L.	70	F	90.1	5.2	AL Amyloidosis, IgM λ
E.P.	54	F	120.5	4.4	AL Amyloidosis, IgM λ
F.P.	66	M	39.4	7.2	AL Amyloidosis, IgM λ MM
	60.45 ± 8.76		69.93 ± 38.36	5.57 ± 3.20	

Data are expressed as mean ± SD. Abbreviations: eGFR, estimated glomerular filtration rate (CKD-EPI equation); uProt, proteinuria; LCDD, light chain deposition disease; MM, multiple myeloma; mMM, non-secretory MM; FibGNF, fibrillary glomerulonephritis.

**Table 3:** Adverse reactions/toxicity of agents currently employed for treatment of MGRS/MM.

Major Regimens/Associations	Renal Toxicities
Alkylating agents	
Melphalan	AKI
Cisplatin	ATN > Fanconi syndrome > TMA
Cyclophosphamide	Haemorrhagic cystitis, SIAD
Anthracyclines	Glomerulonephritis, proteinuria
Proteasome inhibitors	
Bortezomib	TMA > interstitial nephritis
Carfilzomib	TMA > hypertension (vasoconstrictor effects)
Immunomodulators	
Thalidomide	↑ serum creatinine
Lenalidomide	variable, ↑ serum creatinine, hypokalemia
Pomalidomide	AKI, crystal nephropathy
BRAF inhibitors	
Vemurafenib	AKI > interstitial nephritis
Dabrafenib	AKI > interstitial nephritis
SLAMF7 antagonists	
Elotuzumab	AKI
AKT/MTOR inhibitors	
Perifosine	Hypophosphatemia
Rapamycine	Proteinuria, rare AKI
Everolimus	Proteinuria, rare AKI
Anti-IL6 MAb	
Siltuximab	Hyperuricemia, hyperkalemia
Anti PD-1 immune checkpoint inhibitors	
Nivolumab	AKI > interstitial nephritis
Pembrolizumab	AKI > Interstitial nephritis
Anti-KIR agents	
Lirilumab	AKI, hyperuricemia

Abbreviations: AKI: acute kidney injury; ATN: acute tubular necrosis; TMA, thrombotic micro-angiopathy; SIAD, inappropriate antidiuresis syndrome; BRAF, B-homologue Raf murine sarcoma viral oncogene; SLAMF7, signaling lymphocytic activation molecule F7; AKT, serine-threonine protein kinase B; mTOR, mammalian target of rapamycin; PD-1, programmed cell death-1; KIR, killer Ig receptor. ↑, increased.

**Table 4:** Free light chain (FLCs) levels before and after 5 HFR sessions in 3 pts with κ LC and 2 pts with λ LC nephropathies. Post-treatment levels are corrected for ultrafiltration. Two patients recovered from HD in the κ LC group vs. 1 λ LC patient. Data are averaged from 5 HFR sessions in each individual patient, pre-(baseline), and post-(after) 4 h HFR sessions ± SEM, one-way ANOVA on paired observations.

	Kappa FLC (mg/dL)	Lambda FLC (mg/dL)
Baseline	5599.6 ± 672.0	4176.8 ± 279.0
After HFR	2987.1 ± 515.1	1603.2 ± 151.8
*n* of patients	3	2
% removal	46.5 ± 4.5	60.2 ± 19.0
*p*	<0.002	<0.0001
